# Xanthine oxidase inhibitor urate-lowering therapy titration to target decreases serum free fatty acids in gout and suppresses lipolysis by adipocytes

**DOI:** 10.1186/s13075-022-02852-4

**Published:** 2022-07-25

**Authors:** Monica Guma, Benyamin Dadpey, Roxana Coras, Ted R. Mikuls, Bartlett Hamilton, Oswald Quehenberger, Hilda Thorisdottir, David Bittleman, Kimberly Lauro, Shannon M. Reilly, Ru Liu-Bryan, Robert Terkeltaub

**Affiliations:** 1 Department of Medicine, UC San Diego, San Diego VA Healthcare Service, 3350 La Jolla Village Drive, San Diego, CA 92161 USA; 2 Department of Medicine, Autonomous University of Barcelona, Plaça Cívica, 08193 Bellaterra, Barcelona Spain; 3 Division of Metabolism and Endocrinology, Department of Medicine, University of California-San Diego, La Jolla, CA 92093 USA; 4 University of Nebraska Medical Center, Omaha, NE 68198 USA; 5 Weill Center for Metabolic Health, Department of Medicine, Weill Cornell Medicine, New York, NY 10021 USA

**Keywords:** Xanthine oxidase, Lipolysis, Metabolomics, Gout, Adipocytes, Microbiome

## Abstract

**Objective:**

Linked metabolic and cardiovascular comorbidities are prevalent in hyperuricemia and gout. For mechanistic insight into impact on inflammatory processes and cardiometabolic risk factors of xanthine oxidase inhibitor urate-lowering therapy (ULT) titration to target, we performed a prospective study of gout serum metabolomes from a ULT trial.

**Methods:**

Sera of gout patients meeting the 2015 ACR/EULAR gout classification criteria (*n* = 20) and with hyperuricemia were studied at time zero and weeks 12 and 24 of febuxostat or allopurinol dose titration ULT. Ultrahigh performance liquid chromatography-tandem mass spectroscopy acquired the serum spectra. Data were assessed using the Metabolon and Metaboloanalyst software. Lipolysis validation assays were done in febuxostat and/or colchicine-treated 3T3-L1 differentiated adipocytes.

**Results:**

Serum urate decreased from time zero (8.21 ±1.139 SD) at weeks 12 (5.965 ± 1.734 SD) and 24 (5.655 ±1.763 SD). Top metabolites generated by changes in nucleotide and certain amino acid metabolism and polyamine pathways were enriched at 12 and 24 weeks ULT, respectively. Decreases in multiple fatty acid metabolites were observed at 24 weeks, linked with obesity. In cultured adipocytes, febuxostat significantly decreased while colchicine increased the lipolytic response to β-adrenergic-agonism or TNF.

**Conclusion:**

Metabolomic profiles linked xanthine oxidase inhibitor-based ULT titration to target with reduced serum free fatty acids. In vitro validation studies revealed that febuxostat, but not colchicine, reduced lipolysis in cultured adipocytes. Since soluble urate, xanthine oxidase inhibitor treatment, and free fatty acids modulate inflammation, our findings suggest that by suppressing lipolysis, ULT could regulate inflammation in gout and comorbid metabolic and cardiovascular disease.

**Supplementary Information:**

The online version contains supplementary material available at 10.1186/s13075-022-02852-4.

## Introduction

Hyperuricemia, defined by a level of soluble urate that surpasses the solubility threshold of uric acid of 6.8 or 7.0 mg/dL in physiologic solution, has greater than 20% prevalence in the USA [1]. Hyperuricemia is a prerequisite for gout, and it is strongly associated with diseases including hypertension, chronic kidney disease, atherosclerosis, metabolic syndrome, and type 2 diabetes that are very common comorbid conditions in gout patients [2–4]. Soluble urate does not simply appear to behave as an inert end product of purine metabolism [5, 6]. Independent of promoting tissue monosodium urate crystal deposition in gout, and uric acid and calcium oxalate nephrolithiasis [7], excess soluble urate has been observed to promote oxidative stress, inflammation, and vasoregulatory actions that may contribute to cardiovascular and metabolic diseases [5, 8–10]. At the cellular level, the effects of excess soluble urate include perturbation of nitric oxide metabolism [11] and of the renin-angiotensin axis [12], promotion of insulin resistance mediated partly via inhibition of hepatic AMP-activated protein kinase (AMPK) [13], and a role in nonalcoholic steatohepatitis (NASH) through the stimulation of lipogenesis and the inhibition of fatty acid (FA) oxidation [14–17].

Xanthine oxidase inhibitor (XOI) therapy, particularly by use of allopurinol and febuxostat, is the primary approach used to treat hyperuricemia and thereby lower body urate stores [18]. XOI has had favorable treatment effects on experimental small animal models of hypertension, renal disease progression, atherosclerosis, and NASH [16, 19, 20], though net effects of XOI-based urate-lowering therapy remain unclear in adult humans with these conditions [21].

Metabolomics can provide valuable assessment of metabolic effects of specific disease treatments on pathogenic factors [22]. Hence, we seminally examined gout patient metabolic profiles in response to XOI-based ULT titration to target, examining sera in a prospective, randomized clinical trial cohort from a comparative effectiveness of allopurinol and febuxostat [23]. The results provide new mechanistic insight into association of metabolic and cardiovascular comorbidities with gout and hyperuricemia.

## Methods

### Subjects

At the San Diego Veterans Affairs Healthcare Service (SDVAHCS) research site, we conducted a prospective study ancillary to the national, multi-site comparative effectiveness ULT trial VA CSP594 STOP GOUT, in which gout patients were randomized to a treat to target regimen using allopurinol or the more selective XOI febuxostat [23, 24]. Twenty consecutive patients meeting the 2015 ACR/EULAR gout classification criteria [25] with hyperuricemia were recruited from the Rheumatology Outpatient Clinic at SDVAHCS. We characterized gout patient metabolic profiles at time zero and 12 and 24 weeks of treat to target ULT to attempt to achieve serum urate target < 6 mg/dL, done in a blinded way for XOI used and following the trial protocol [23, 24]. The clinical trial and ancillary study were approved by the VA Institutional Board Review, and all subjects signed an informed consent for both studies. A second set of patients recruited at University of Nebraska Medical Center, in Omaha, NE, were the source of serum samples for lipidomic profiling of serum free fatty acids.

All those studied had a clinical assessment by the study physician for palpable tophaceous disease and presence of active flare or quiescent arthritis, and co-morbidities and current medications were recorded. Research personnel collected non-fasting blood samples into 10 ml BD Vacutainer Blood Collection Tubes containing spray-coated silica and a polymer gel for serum separation. After 30 min incubation at room temperature, tubes were centrifuged for 10 min at 2000×g, and sera were transferred into 1.7 ml tubes and immediately frozen and stored at − 80 °C until analysis.

### Cell culture—3T3-L1 adipocytes

3T3-L1 fibroblasts (American Type Culture Collection) were cultured in culture media (DMEM containing 4.5 g/l glucose, 10% FBS, 10 U/ml penicillin, 10 U/ml streptomycin, and 292 mg/l glutamine). Once grown to confluence, adipocyte differentiation was initiated using a three-component cocktail containing 500 μM 3-isobutyl-1-methylxanthine, 250 nM dexamethasone, and 1 μg/ml insulin for the first 3 days, followed by an additional 4 days of media containing 1 μg/ml insulin, and finally, differentiation was completed in the culture media. Only cultures, in which > 90% of cells displayed adipocyte morphology, were used. Fully differentiated adipocytes which were cultured in culture media for 7–9 days were used for experiments. TNF was added to the culture media at 17 mg/ml, 36 h prior to assaying lipolysis. Cells were pretreated with 50 μM febuxostat and/or 10 nM colchicine in complete media for 72 h prior to assaying lipolysis, the media, and was replenished every 24 h during this pretreatment.

### Lipolysis assay

Pretreated fully differentiated 3T3-L1 cells were washed with PBS prior to incubating with DMEM with 2% FFA free BSA in the presence or absence of 10 μM CL-316,243 (a highly selective β-3 adrenergic receptor agonist). Media was collected after 30 or 60 min, and the FFA content in the media was measured using the Wako HR series NEFA-HR assay (Catalog No. 999-34691, 995-34791, 991-34891, 993-35191, 276-76491) following manufacturer protocol.

### Selective plasma free fatty acid panel analysis

Aliquots of 20uL human serum were extracted by a bi-phasic solution of acidified methanol and isooctane, derivatized using PFBB, and analyzed by gas chromatography–mass spectrometry (GC-MS) on an Agilent 6890N gas chromatograph equipped with an Agilent 7683 autosampler. Fatty acids were separated using a 15 m ZB-1 column (Phenomenex) and monitored using SIM identification. Analysis was performed using the Mass Hunter software [26]. Concentrations are reported in pmol/mL.

### Metabolon platform

The details for the Metabolon platform are in the supplementary data.

### Statistical analysis

Principal component analysis (PCA) along with hierarchical clustering analysis (HCA) as well as random forest (RF) analysis and two-way repeated measures ANOVA was performed using the Metabolon software. We employed MetaboAnalyst version 5.0, an open resource, for the rest of metabolomics analysis [27, 28]. Partial least squared discriminant analyses (PLS-DA) to identify discriminant metabolites controlling for multicollinearity, and cross-validation accuracy and permutation model statistics were retrieved. Pathway analysis was evaluated with the tool of enrichment analysis available on MetaboAnalyst, using the set KEGG 2019, which contains 84 metabolite sets [29]. Other statistical analysis was performed with the SPSS software version 26.0. Continuous variables were expressed as mean ± standard deviation (SD) or standard error of mean (SEM) and categorical variables as percentage. In vitro experiments with adipocytes were analyzed by Holm-Sidak post hoc test after significant 2-way ANOVA. Results were considered significant if the 2-sided *p* value was less than 0.05.

## Results

### Patient demographics and disease characteristics

We recruited 20 male subjects meeting the 2015 ACR/EULAR gout classification criteria, with mean age 60.4 years (11.1) and mean body mass index (BMI) of 31.5 (4.4). All gout subjects had hyperuricemia (serum UA 7.0-11.3 mg/dL (8.34 ± 1.2SD) at time zero, and 45% patients (9 out of 20) had flare rate ≥ 5/year. Demographics of the patients along with the disease characteristics, comorbidities, and treatment they were receiving are summarized in Table 1. Six subjects entered the study on allopurinol, but at doses where they still were hyperuricemic and therefore not at serum urate target; these subjects were randomized to febuxostat 40 mg/day or allopurinol and were studied on further XOI dose titration, per protocol [23], to serum urate target of < 5.0 mg/dL for disease with palpable tophi and < 6.0 mg/dL where no palpable tophi were detected by the study physician. The remaining 14 patients started ULT titration to target, with allopurinol or febuxostat at recruitment and underwent XOI-based ULT titration to target. Serum urate levels at week 12 (mean 5.97±1.7 SD, range 4–8.5) and week 24 (mean 5.66±1.7 SD, range 3.5–9) were significantly lower compared to time zero (mean 8.21±1.14 SD, range 6.8–10.2; *p* < 0.05 both time points), and 80% and 90% patients achieved reduced serum UA to < 7 mg/dL at week 12 and week 24, respectively, via ULT titration. Patients did not change any other concomitant treatments (including statins and antidiabetic medications) or undergo dietary change for weight loss during the 24 weeks of the study.

**Table 1:** Demographics of *n* = 20 patients with gout at baseline

	Baseline
**Age**	60.4 ± 11.1
**Gender**	100% M
**Race**	7 W, 5 AA, 3 A, 4 PI, 1 NA
**DM (*n*)**	7 (4 on metformin, 3 on insulin)
**DL (*n*)**	15 (10 on statins)
**HBP (*n*)**	18
**Flares per year**	4.48 ± 3.35
**Uric acid**	8.34 ± 1.2
**BMI**	31.7 ± 4.4
**ALT**	31.6 ± 13
**AST**	38.1 ± 30.1
**TG**	225.94 ± 124.4
**LDL**	94.1 ± 34.8

*M* male, *W* white, *AA* African American, *A* Asian, *PI* pacific-islanders, *NA* native-American, *DM* diabetes mellitus, *DL* dyslipidemia, *HBP* high blood pressure, *BMI* body mass index, *TG* triglycerides, *LDL* low-density lipoproteins

### Metabolomic profiling at time zero

**Fig. 1:**
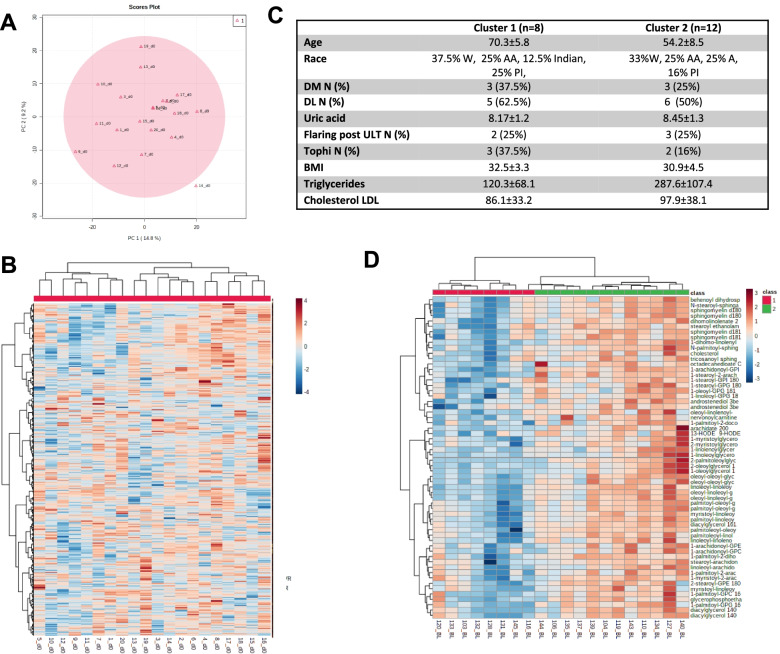
Metabolomic profiling at time zero. **A** PCA examining samples at time zero. **B** Heat map based on Pearson and Ward for determining distance and clustering identified 2 clusters at time zero. **C** Characteristics of patients in both clusters. Continuous variables were expressed as mean ± standard deviation (SD) and categorical variables as number (*N*) and percentage. **D** Top 50 metabolites different between clusters

Mass spectrometry identified 1105 compounds of known identity (named metabolites and listed in supplementary excel file). We first conducted principal component analysis (PCA). At time zero, samples showed spread among components 1 and 2, suggesting that subjects enrolled in this study varied in their serum metabolome (Fig. 1A). Heat map and K-cluster analysis identified 2 clusters at time zero (Fig. 1B). These two groups were clustered by the concentration of triglycerides in plasma (120.3 ± 68.1 vs 287.6 ± 107.4, *p* < 0.01) and age (70.3 ± 5.8 vs 54.2 ± 8.5, *p* < 0.01), but not for clinical outcomes (Fig. 1C, D). Partial least squares discriminant analysis (PLS-DA) and random forest analysis confirmed the expected discrimination and prediction when subjects were analyzed by BMI for obesity compared to non-obesity. By contrast, number of flares (> 5/year vs < 5/year), uric acid levels at time zero (HU > 8 versus HU < 8), or the presence of 1 or more palpable tophi failed to separate the metabolic profiles of the gout patients (Supplementary Fig. 1).

### Validation of XOI-based ULT effects on xanthine and purine metabolism by serum metabolomic profiling

**Fig. 2:**
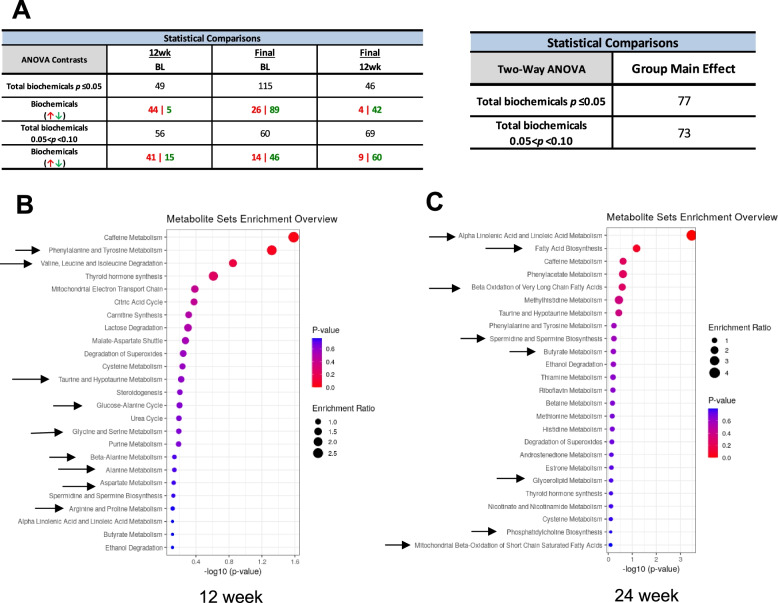
XOI-based ULT effects on serum metabolomic pathway analysis. **A** Two-way repeated measures ANOVA identified 115 metabolites (89 decreased and 26 increased) significantly differing between baseline and 24 weeks ULT titration to target. A summary of the numbers of metabolites that achieved statistical significance (*p* ≤ 0.05), as well as those approaching significance (0.05 < *p* < 0.10), is shown. **B**, **C** Pathway analysis was conducted with the 105 metabolites (*p* < 0.1) significant at 12 weeks ULT (**B**) and with the 165 metabolites (*p* < 0.1) at 24 weeks (**C**). At 12 weeks ULT, pathways were significantly enriched with changes in the AA metabolism (arrows, **B**). However, at 24 weeks ULT, significant altered pathways were mostly related to FA and polyamine metabolism (arrows, **C**)

PCA analysis with all the samples demonstrated overlap between samples collected at time zero and at 12 and 24 weeks ULT titration to target (Supplementary Fig. 2A). Since sera at 12 and 24 weeks did not have significant separation between the groups, length of ULT titration to target did not appear to contribute to separation and differences between groups (Fig. 2A). Hierarchical clustering analysis showed clustering based on the subject (as suggested in Fig. 1A) but not treatment itself (Supplementary Fig. 2B). Yet, random forest analysis, using metabolite data derived from serum samples collected at time zero or 12 and 24 weeks ULT titration to target, identified several metabolites contributing most to the separation between time zero and 24 weeks of ULT titration to target. Top metabolites identified by this analysis highlighted changes in selected metabolic pathways (Supplementary Fig. 2C), resulting in predictive accuracy of 52% (compared to 33% expected by random chance alone) (Supplementary Fig. 2D). As expected, top serum metabolites generated by random forest analysis also included members of amino acid (AA) and FA metabolism pathways (Supplementary Fig. 2C). We validated that samples collected from subjects undergoing XOI-based ULT titration to target showed significant alterations in allopurinol and febuxostat and in xanthine and purine metabolism at 12- and 24-week treatment (Supplementary Fig. 3). Sera at 12- and 24-week XOI-based ULT titration to target had both significantly lower levels of urate compared to time zero and increases in the uric acid precursor, xanthine and xanthosine, and other methyluric acid and methylxanthine changes that validated perturbation of purine metabolism (Supplementary Fig. 3).

### Alterations in AA metabolism pathways at 12-week XOI-based ULT titration to target

Using two-way repeated measures ANOVA, we identified 115 metabolites (89 decreased and 26 increased) significantly differing between time zero and 24 weeks of ULT titration to target. Figure 2A summarizes the numbers of metabolites that achieved statistical significance (*p* ≤ 0.05) and those approaching significance (0.05 < *p* < 0.10). Pathway analysis was conducted with the 105 metabolites (*p* < 0.1) significant at time 12 weeks (Fig. 2B) and with the 165 metabolites (*p* < 0.1) at time 24 weeks ULT titration to target (Fig. 2C). Notably, at 12 weeks of ULT titration to target, significant metabolic pathways were enriched in AA metabolism. However, at 24 weeks of XOI-based ULT titration to target, significant metabolic pathways were mostly related to FA and polyamine metabolism.

**Fig. 3:**
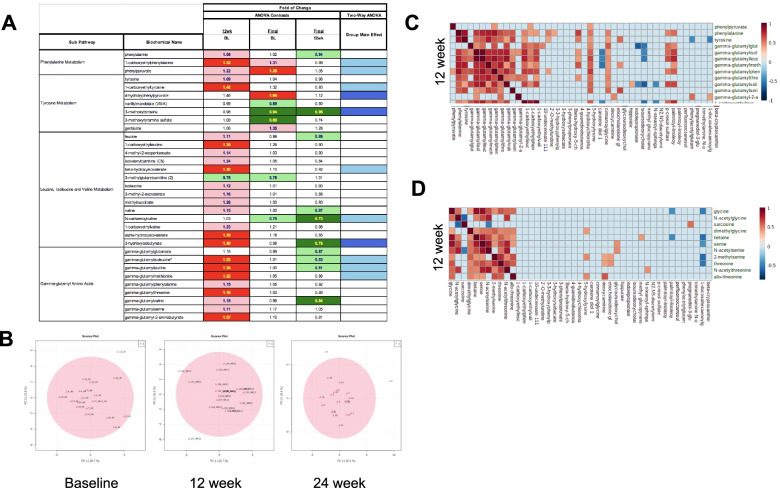
XOI-based ULT effects on AA metabolism. **A** Samples collected specially at 12 and 24 weeks of treatment showed significant alterations in aromatic AA, branched-chain AA, G-glutamyl AA. Green: indicates significant difference (*p* ≤ 0.05) between the groups shown, metabolite ratio of < 1.00. Light green: narrowly missed statistical cutoff for significance 0.05 < *p* < 0.10, metabolite ratio of < 1.00. Red: indicates significant difference (*p* ≤ 0.05) between the groups shown, metabolite ratio of ≥ 1.00. Light red: narrowly missed statistical cutoff for significance 0.05 < *p* < 0.10, metabolite ratio of ≥ 1.00. Blue: indicates significant (*p* ≤ 0.05) ANOVA. Light blue: indicates 0.05 < *p* < 0.10 ANOVA effect. **B** PCA at different time points using only microbiome-related metabolites. **C** Correlation between metabolites derived from AA that were significant at 12 weeks of ULT titration to target (*Y*-axis) and metabolites shown to predict microbiome diversity (*X*-axis). **D** Correlation between metabolites derived from AA that were not significant at 12 weeks of ULT titration to target (*Y*-axis) and metabolites shown to predict microbiome diversity (*X*-axis). Pearson correlation (*r*) in **C** and **D** with a cutoff value of 0.5. The orange color indicates a positive correlation > 0.5, and the dark blue indicates negative correlation ≤0.5

At 12-week ULT, most metabolites that contributed robustly to group discrimination were relevant to AA metabolism. These included derivatives of the aromatic AAs, phenylalanine and tyrosine, and derivatives of branched-chain AA, leucine, isoleucine, and valine. In addition, we observed widespread increases in subsets of gamma-glutamylated AAs—gamma-glutamyl (GG)-leucine, GG-isoleucine, GG-threonine, GG-valine, and GG-phenylalanine—in sera at 12 weeks ULT compared to samples from time zero (Fig. 3A).

Gut dysbiosis is involved in several metabolic diseases including diabetes, obesity, and NASH [30–32] and has been suggested to potentially link gout to comorbid metabolic disease [33, 34]. Gut microbiota are actively involved in both aromatic and branched-chain AA metabolism [35], and the human gut microbiome is involved in deconjugation of human primary bile acids and their subsequent biotransformation to secondary bile acids [36]. In this context, secondary bile acids were also altered at 12 weeks ULT titration to target (Supplementary Fig. 4A). To determine whether such changes observed at 12 weeks could be related to changes in the microbiome metabolism induced by XOI-based ULT titration to target, we studied a group of metabolites that were shown to predict gut microbiome alpha diversity in humans [37]. Figure 3B shows different spread among components 1 and 2 at the three time points when using only these metabolites, and Supplementary Fig. 4B shows that these metabolites correlate differently at the three time points studied, suggesting that ULT titration to target affects the levels of these metabolites and can potentially act indirectly by modulating microbial diversity. In addition, as shown in Fig. 3C, correlation between metabolites derived from AA that were significant at 12 weeks of ULT titration to target (*Y*-axis) and metabolites shown to predict microbiome diversity (*X*-axis) was significant but not with metabolites derived from AA that were not significant at 12 weeks of ULT titration to target (*Y*-axis) (Fig. 3D).

### Alterations in FA metabolism pathways at 24 weeks ULT titration to target

**Fig. 4:**
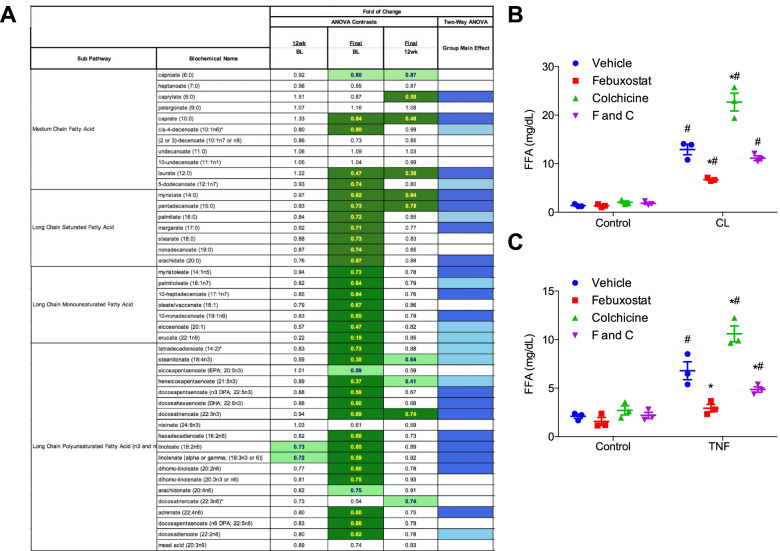
XOI-based ULT effects in lipolysis. **A** Samples collected at 24 weeks of treatment showed significant alterations in medium chain FAs and long chain FAs. Green: indicates significant difference (*p* ≤ 0.05) between the groups shown, metabolite ratio of < 1.00. Light green: narrowly missed statistical cutoff for significance 0.05 < *p* < 0.10, metabolite ratio of < 1.00. Red: indicates significant difference (*p* ≤ 0.05) between the groups shown, metabolite ratio of ≥1.00. Light red: narrowly missed statistical cutoff for significance 0.05 < *p* < 0.10, metabolite ratio of ≥ 1.00. Blue: indicates significant (*p* ≤ 0.05) ANOVA. Light blue: indicates 0.05 < *p* < 0.10 ANOVA effect. **B** Free FA release into the media over 30 min following administration of 10 μM CL-316,243 [32] or vehicle control to 3T3-L1 adipocytes pretreated with febuxostat (*F*, 50 μM) and/or colchicine (*C*, 10 nM) for 72 h. **C** TNF treatment at 17 ng/mL was administered for 36 h before the addition of febuxostat (50 μM) and/or colchicine 10 nM. Lipolysis was assessed 72 h later by measuring free FA secreted into the cell culture media over 60 min. Data in **B** and **C** presented as mean ± SEM. ^#^*p* < 0.05, by Holm-Sidak post hoc test after significant 2-way ANOVA for the control versus CL (**B**) or TNF (**C**) treated groups, within vehicle, F, C, or F and C treated group. *p < 0.05, by Holm-Sidak post hoc test after significant 2-way ANOVA for the vehicle versus F, C, or F and C treated groups, within CL (**B**) or TNF (**C**) treated group

At 24 weeks of the XOI-based ULT titration to target, most of the metabolites that contributed robustly to group discrimination were FA species. Medium-, long saturated-, long monounsaturated-, and long polyunsaturated FA were significantly decreased in samples collected at 24 weeks ULT compared to time zero (Fig. 4A and Supplementary Excel file). This occurred despite lack of prescribed diet change or of decreased body weight during the length of this study.

Adipocyte lipolysis is an import source of serum FA [38]. During lipolysis, triglycerides are broken down into FAs and glycerol. Consistent with reduced adipocyte lipolysis, glycerol was also decreased by ULT (Fig. 4A). To validate this mechanism, we next assessed if XOI could directly regulate lipolysis in adipocytes. Treatment of cultured adipocytes with febuxostat, but not colchicine, resulted in significantly decreased intracellular uric acid by~ 50%, as compared to no significant change with colchicine (data not shown) Furthermore, febuxostat reduced the rate of adipocyte lipolysis stimulated by activation of β-adrenergic signaling by the β-3 adrenergic receptor agonist CL-316,243 (Fig. 4B). Inflammatory signals such as TNF are also known to increase adipocyte lipolysis [39, 40]. Febuxostat but not colchicine pre-treatment also blocked the stimulation of lipolysis by TNF (Fig. 4C). Thus, suppression of adipocyte lipolysis appeared to be at least one mechanism by which febuxostat treatment resulted in lower serum FA and glycerol levels in the patient samples. Other pathways altered at 24 weeks ULT that could impact inflammation were serum vitamins (Supplementary Fig. 5A) and polyamines (Supplementary Fig. 5B).

### Free fatty acid (FFA) panel assessment by lipidomics at 24 weeks ULT titration to target in a validation cohort

**Fig. 5:**
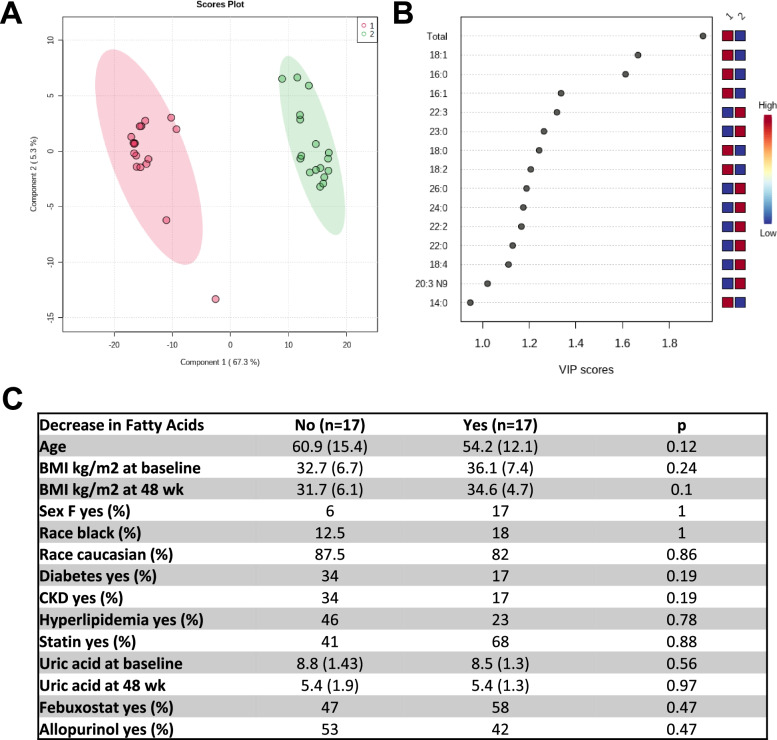
XOI-based ULT effects on FA metabolism. **A** PLS-DA separation between patients with different pattern of FA levels after ULT therapy: 17 patients did not decrease FA [1], and 17 patients significantly decreased FA [2]. **B** The variables important in projection (VIP) in discriminate both groups, where a VIP score ≥ 1 was considered as important. **C** Characteristics of patients in both clusters. Continuous variables were expressed as mean ± standard deviation (SD) and categorical variables as number (*N*) and percentage

Plasma FFA were analyzed in a validation cohort from the University of Nebraska Medical Center. Thirty-four subjects (4 women and 30 men) meeting the 2015 ACR/EULAR gout classification criteria, with mean age 57.2 years (14.1), mean body mass index (BMI) of 34.1 (7.1), and mostly White. All gout subjects had hyperuricemia (8.67 ± 1.3) at time zero. We only detected three FA (12:0, 22:0, and 22:1) that were decreased after 24 weeks of ULT. Of interest, K-cluster and PLS-DA analysis identified 2 clusters (Fig. 5A). These two groups were clustered by the changes in FA levels after 24 weeks of ULT (17 patients with a decrease in FA levels vs 17 patients that did not have a decrease in FA levels after 24 weeks of ULT, Supplementary Excel file and Fig. 5B), but not by clinical outcomes (Fig. 5C). We then reanalyzed the first cohort from San Diego, and we detected a small group of 5 patients (out of 20) that did not have a decrease of FA after 24 weeks of ULT (Supplementary Excel file). Of note, these 5 patients were mostly Caucasian (80% vs 20%, in patients without decrease of FA versus patients with significantly decrease of FA respectively, *p* = 0.05) and leaner (BMI of 28.7 ± 0.7 vs 32.3 ± 4.3, *p* < 0.01, in patients without decrease of FA versus patients with significantly decrease of FA respectively) than the group with significant decreased FA levels.

## Discussion

In this study, we gained new insight into downstream effects of XOI-based ULT titration to target on metabolism, using a prospective, untargeted approach to determine gout patient metabolic profiles. XOI-based ULT titration to target was associated with significant changes in AA metabolism between time zero and 12 weeks of ULT treatment, specifically in phenylalanine, tyrosine, and branch-chain AA metabolism. Proteolysis in the gastrointestinal tract generates AA that are actively sensed and processed by both the host and microbiota [41]. Most proteins and peptides from dietary origins normally undergo digestion in the small intestine and get absorbed [41]. Proteins that escape digestion in the small intestine are present in the colonic lumen, where they serve as fermentable substrates for the gut microbiota and undergo intense proteolysis into AA [41]. Notably, gut microbiota is actively involved in both aromatic and branched-chain AA metabolism [35]. A previous report identified differential fecal AA between gout patients and healthy controls [33]. Here, XOI-based ULT titration to target appeared to alter not only selective metabolism of AA but also gamma-glutamylation itself. In this light, gamma-glutamyl AA are produced when gamma-glutamyl transpeptidase (GGT) catalyzes the transfer of the gamma-glutamyl moiety of glutathione to AAs. GGT is present in some mammalian tissues, most notably the liver, and also in several bacterial species [42].

Our results raised important questions on whether changes observed in AAs and gamma-glutamylation, associated with the ULT approach employed, were mediated by changes secondary to effect of the ULT on the microbiome and/or due to decreased serum urate concentration. Prior studies have suggested that intestinal microbiota distinguished gout patients from healthy humans [33, 34]. Hence, ULT could help to restore more homeostatic microbiome. In this regard, a prior study in rats showed the relationship between hyperuricemia and microbiome [43]. A high purine diet affected the rat gut microbiome, and circulating urate levels decreased in hyperuricemic rats fed with antibiotics [43]. Moreover, transfer of gut microbiome from hyperuricemic rats to wild-type rats increased serum urate levels in recipient rats, suggesting a feedback loop between circulating levels of urate and microbiome diversity [43]. Another study demonstrated that allopurinol caused unique changes in microbiome genera in male rats with hyperuricemia [44].

Since branched chain AA, phenylalanine, and tyrosine play critical roles in the regulation of energy homeostasis, nutrient metabolism, gut health, and immunity [45], metabolic changes induced by XOI-based ULT titration to target could be regulating metabolism of glucose, lipid, and proteins, and inflammation via these AA, while decreasing uric acid. At a cellular level, several mechanisms have been proposed to explain the effect of uric acid on metabolism and inflammation. Modulation of AMPK-mTOR [45] and phosphatidylinositol 3-kinase (PI3K)-AKT signaling pathways [45] in adipose, skeletal muscle, and immune cells are some of the mechanisms proposed. Metagenomic studies will be needed to dissect the potential direct role of ULT in microbial changes and function and effects of such alterations in the observed metabolomic changes. In our cohort, changes in AA metabolism were not observed at 24 weeks treatment, suggesting that microbiome changes could be transient, a finding potentially buttressed by prior work revealing that most shifts observed in the microbiome after environmental insults are temporary [46].

XOI-based ULT titration to target for 24 weeks was associated with significant changes in metabolic pathways mostly related to FAs and polyamine metabolism. Not all FA subtypes were equally altered, since medium and long chain FAs were significantly decreased, but not FA derivatives (e.g., acyl-glutamine, acyl-glycine or acyl-carnitine). And not all patients in both cohorts had this significant decrease of circulating FA levels. This observation is consistent with a change in lipolysis, which releases FAs, but not FA derivatives, into the blood stream. The lack of uniform alteration in serum FAs also aligns with inconclusive data on the effects of ULT on blood lipids in previous reports. The association between gout and dyslipidemia is partly genetically mediated [47] but is otherwise not well understood. Differences in race and ethnicity and baseline BMI could explain the heterogeneity of the results. Moreover, effects of ULT on blood triglycerides and cholesterol have been inconclusive both in animal experiments and clinical studies [21, 48–50]. Given the complexity of lipid metabolism, measuring triglycerides and cholesterol may not be able to capture subtle differences or the effect of ULT on specific classes of lipids.

Liver and adipose tissue are the main tissues involved in the metabolism of lipids [38]. Furthermore, both uric acid metabolism and XOI-based ULT clearly impact liver metabolism and NASH. Specifically, hepatocellular increase in urate has been linked with the pathogenesis of fatty liver disease via both stimulation of lipogenesis and inhibition of FA oxidation [14, 15]. Soluble urate also has been reported to directly induce hepatocyte fat accumulation by activating the NLRP3 inflammasome; conversely, lowering uric acid production by allopurinol inhibited NLRP3 inflammasome activation in a high fat diet mouse model of NAFLD [51]. Additional reports have supported a direct effect of soluble urate on hepatocyte lipid accumulation [15, 52]. It remains unclear why febuxostat, but not allopurinol, exerted beneficial effects in high-fat, high-cholesterol, and high-cholate dietary model of murine steatohepatitis [15].

Adipocytes play a vital role in regulating FA homeostasis [38]. However, the impact of purine and uric acid metabolism is incompletely defined in adipocytes. This cell type expresses xanthine oxidase, releases urate as well as free FA, and known effects of uric acid metabolism and related oxidative stress modulate adipocyte differentiation [53]. In prior work, adipocytes from the white adipose tissue of patients with hyperuricemia were hypertrophied, and in vivo and in vitro studies reported the links between uric acid and FA metabolism [54]. Here, observing not only decreases in multiple serum free FA levels, but that serum glycerol was also decreased by XOI-based ULT titration to target, we focused on studying the rate of adipocyte lipolysis in response to XOI treatment in culture. The highly selective XOI enzyme channel inhibitor febuxostat [55], rather than the much less selective XOI substrate inhibitor drug allopurinol, was chosen for these experiments. Since patients in the clinical trial used for the ancillary study were on daily colchicine for much of the first 24 weeks, and prior studies suggested a role of colchicine in FA metabolism and modulation of the gut microbiome [56, 57], we investigated the effect of colchicine on lipolysis *in vitro*. Febuxostat, but not colchicine, significantly decreased lipolysis by adipocytes.

The discoveries herein that XOI-based ULT titration to target decreases multiple free FA in gout subjects, and that febuxostat decreases lipolysis by adipocytes have several potentially important implications for patients with gout. First, increased lipolysis is a major pathogenic factor in insulin resistance, type IV hyperlipidemia, and the broader phenotype of metabolic syndrome and obesity [58, 59]. Specifically, lipolysis modulates visceral fat and affects hepatic metabolism, glucose production and synthesis of very low-density lipoprotein, and also promotes decrease in HDL [60]. High levels of circulating free FA and impaired insulin activity promote hyperglycemia, not only by increased glucose production by the liver but also by reduced glucose uptake by muscle and adipose tissue, and also may contribute directly to NASH [61]. In addition, lipolysis can modulate inflammation, inducing chronic low grade metabolic inflammation [62] and engaging receptors on the cell surface or stress kinases within the cytoplasm. Free FA such as palmitate can directly activate inflammatory pathways in several cell types by increasing TLR4 signaling [36] and by stimulating signaling molecules such as PKR and JNK, triggering the secretion of inflammatory mediators [63]. Taken altogether, the suppression of lipolysis by XOI-based ULT could ameliorate inflammation in gout patients, reducing their risk of comorbid metabolic and cardiovascular diseases.

Limitations of this metabolomic study include the relatively small number of subjects; further replication will be valuable in clinical trials going forward. Because this study was seminal, and small in population size, we did address study limitations by using subjects as their own controls and studying each subject at 3 time points (0, 12, and 24 weeks) over the course XOI-based ULT titration to target. Importantly, we also validated that patients clustered at time zero per BMI and hypertriglyceridemia and that the XOI-based ULT impacted purine and xanthine metabolism. Other inherent limitations, not addressed directly in our study design, include the non-fasting samples, the use of only XOI drugs, and not uricosurics or uricase therapy, to treat hyperuricemia in the parent comparative effectiveness clinical trial. In addition, we cannot rule out potential confounding effects of comorbidities or of changes in gouty arthritis activity over the course of the first 24 weeks ULT titration to target. Our results also do not rule out an additional contribution of the liver to the results obtained. Gut-liver axes involving metabolism and the microbiome are well described [30–32]. In addition, increased intestinal permeability has been detected in hyperuricemic mice [64]. Increased LPS and TNF levels in hyperuricemic mice have suggested the possibility that hyperuricemia, at least in mice, induces a state of low-grade systemic inflammation that could modify lipid metabolism in the liver [64].

## Conclusions

In conclusion, serum profiles linked with patient response to XOI-based ULT titration to target in this seminal, prospective analysis indicated multiple changes in metabolism related to treatment, including alterations of serum levels of AA, polyamines, serum vitamins, and FAs, that modulate inflammation, and could impact gouty arthritis and multiple gout-associated comorbid conditions including obesity, metabolic syndrome, type II diabetes mellitus, NAFLD, and atherosclerosis. Further studies are warranted to investigate how urate and XOI treatment modulates AA and FA metabolism in adipose tissue, the gut, and the liver. Our findings suggest that decreased lipolysis by adipocytes, and consequent associated decrease in multiple sera free FA levels in response to XOI-based ULT titration to target in gout, could modulate gouty arthritis and several comorbid metabolic and cardiovascular diseases in gout patients.

## Supplementary Information

**Additional file 1: Supplementary Figure 1**. PLS-DA and RF analysis of serum metabolomic profiling at time zero. PLS-DA and RF analysis at time zero resulted in a good discrimination and prediction of the samples per BMI (A), but not per number of flares (B), or hyperuricemia (HU) > 8 mg/dL (C), or presence of tophi (D). **Supplementary Figure 2**. XOI-based ULT effects on serum metabolomic profiling. (A) PCA examining samples at time zero as well as at 12 and 24 weeks ULT titration to target. (B) Hierarchical clustering analysis at three time points. (C) Random Forest (RF) analysis using metabolite data derived from sera collected at baseline, or at 12 and 24 weeks ULT titration to target. (D) Top metabolites generated by RF analysis resulted in predictive accuracy of 52% (compared to 33% expected by random chance alone). **Supplementary Figure 3**. Validation of XOI-based ULT effects on xanthine and purine metabolism by serum metabolomic profiling. (A) Levels of ULT drugs included in the treatment and in metabolites related to purine and xanthine metabolism were significantly elevated in samples collected at 12- and 24-weeks treatment. Green: indicates significant difference (*p*≤0.05) between the groups shown, metabolite ratio of < 1.00. Light Green: narrowly missed statistical cutoff for significance 0.05<*p*<0.10, metabolite ratio of < 1.00. Red: indicates significant difference (*p*≤0.05) between the groups shown, metabolite ratio of ≥ 1.00. Light Red: narrowly missed statistical cutoff for significance 0.05<*p*<0.10, metabolite ratio of ≥ 1.00. Blue: indicates significant (*p*≤0.05) ANOVA. Light Blue: indicates 0.05<*p*<0.10 ANOVA effect. (B) Scaled intensity of selected metabolites at the three time points. **Supplementary Figure 4**. XOI-based ULT effects on bile acid metabolism: (A) Samples collected at 12- and 24- weeks of treatment showed significant alterations in bile acid metabolism. Green: indicates significant difference (*p*≤0.05) between the groups shown, metabolite ratio of < 1.00. Light Green: narrowly missed statistical cutoff for significance 0.05<*p*<0.10, metabolite ratio of < 1.00. Red: indicates significant difference (*p*≤0.05) between the groups shown, metabolite ratio of ≥ 1.00. Light Red: narrowly missed statistical cutoff for significance 0.05<*p*<0.10, metabolite ratio of ≥ 1.00. Blue: indicates significant (*p*≤0.05) ANOVA. Light Blue: indicates 0.05<*p*<0.10 ANOVA effect (B) Heatmap and hierarchical cluster analysis indicating positive (on red) and negative (on blue) relationships between metabolites that were recently shown to predict alpha diversity in humans, at different time points. **Supplementary Figure 5**. XOI-based ULT effects on vitamin and polyamine metabolism: (A) Levels of serum vitamins are significantly altered in samples collected at 12- and 24-weeks treatment. (B) Samples collected at 24 weeks treatment showed significant alterations in polyamines. Green: indicates significant difference (*p*≤0.05) between the groups shown, metabolite ratio of < 1.00. Light Green: narrowly missed statistical cutoff for significance 0.05<*p*<0.10, metabolite ratio of < 1.00. Red: indicates significant difference (*p*≤0.05) between the groups shown, metabolite ratio of ≥ 1.00. Light Red: narrowly missed statistical cutoff for significance 0.05<*p*<0.10, metabolite ratio of ≥ 1.00. Blue: indicates significant (*p*≤0.05) ANOVA. Light Blue: indicates 0.05<*p*<0.10 ANOVA effect.

**Additional file 2: Supplementary Table 1**. Included the 1105 compounds of known identity from the Metabolon platform. **Supplementary Table 2**. Relative levels of fatty acids identified in the UCSD cohort. **Supplementary Table 3**. Fatty acids identified in the Omaha cohort. Concentrations are reported in pmol/mL of plasma.

**Additional file 3.** Supplementary methods.

## Data Availability

Data is available upon request to the authors.

## Abbreviations

ULT: Urate-lowering therapy
BMI: Body mass index
FA: Fatty acid
AMPK: AMP-activated protein kinase
NASH: Nonalcoholic steatohepatitis
XOI: Xanthine oxidase inhibitor
SDVAHCS: San Diego Veterans Affairs Healthcare Service
GC-MS: Gas chromatography–mass spectrometry
PCA: Principal component analysis
RF: Random forest
PLS-DA: Partial least squared discriminant analyses
SD: Standard deviation
SEM: Standard error of mean
AA: Amino acid
PI3K: Phosphatidylinositol 3-kinase
