# Supplementary material for: Xanthine oxidase inhibitor urate-lowering therapy titration to target decreases serum free fatty acids in gout and suppresses lipolysis by adipocytes
Source: Arthritis Res Ther. 2022 Jul 25;24:175. doi: 10.1186/s13075-022-02852-4 (PMC9310412; doi:10.1186/s13075-022-02852-4)
Supplement: Supplementary file 1 — Additional file 1: Supplementary Figure 1. PLS-DA and RF analysis of serum metabolomic profiling at time zero. PLS-DA and RF analysis at time zero resulted in a good discrimination and prediction of the samples per BMI (A), but not per number of flares (B), or hyperuricemia (HU) > 8 mg/dL (C), or presence of tophi (D). Supplementary Figure 2. XOI-based ULT effects on serum metabolomic profiling. (A) PCA examining samples at time zero as well as at 12 and 24 weeks ULT titration to target. (B) Hierarchical clustering analysis at three time points. (C) Random Forest (RF) analysis using metabolite data derived from sera collected at baseline, or at 12 and 24 weeks ULT titration to target. (D) Top metabolites generated by RF analysis resulted in predictive accuracy of 52% (compared to 33% expected by random chance alone). Supplementary Figure 3. Validation of XOI-based ULT effects on xanthine and purine metabolism by serum metabolomic profiling. (A) Levels of ULT drugs included in the treatment and in metabolites related to purine and xanthine metabolism were significantly elevated in samples collected at 12- and 24-weeks treatment. Green: indicates significant difference (p≤0.05) between the groups shown, metabolite ratio of < 1.00. Light Green: narrowly missed statistical cutoff for significance 0.05<p<0.10, metabolite ratio of < 1.00. Red: indicates significant difference (p≤0.05) between the groups shown, metabolite ratio of ≥ 1.00. Light Red: narrowly missed statistical cutoff for significance 0.05<p<0.10, metabolite ratio of ≥ 1.00. Blue: indicates significant (p≤0.05) ANOVA. Light Blue: indicates 0.05<p<0.10 ANOVA effect. (B) Scaled intensity of selected metabolites at the three time points. Supplementary Figure 4. XOI-based ULT effects on bile acid metabolism: (A) Samples collected at 12- and 24- weeks of treatment showed significant alterations in bile acid metabolism. Green: indicates significant difference (p≤0.05) between the groups shown, metabolite ratio of [file 13075_2022_2852_MOESM1_ESM.pdf]

Supplementary Figure 1

A BMI

PLS-DA cross validation details:

Measure	1 comps	2 comps	3 comps	4 comps	5 comps
Accuracy	0.75	0.8	0.85	0.85	0.85
R2	0.80488	0.95449	0.99294	0.99904	0.99985
Q2	0.1914	0.26537	0.30242	0.30152	0.30049

The OOB error is 0.15

	2	3	class.error
2	7	2	0.222
3	1	10	0.0909

B Number of flares >5/year

PLS-DA cross validation details:

Measure	1 comps	2 comps	3 comps	4 comps	5 comps
Accuracy	0.45	0.4	0.4	0.4	0.4
R2	0.70887	0.91678	0.99006	0.99789	0.99962
Q2	-0.22122	-0.33119	-0.3816	-0.38436	-0.3731

The OOB error is 0.5

	1	2	class.error
1	8	3	0.273
2	7	2	0.778

C HU>8

PLS-DA cross validation details:

Measure	1 comps	2 comps	3 comps	4 comps	5 comps
Accuracy	0.35	0.4	0.45	0.4	0.45
R2	0.71793	0.92827	0.98931	0.99754	0.99987
Q2	-0.48934	-0.65272	-0.66164	-0.65754	-0.65039

The OOB error is 0.7

	1	2	class.error
1	2	8	0.8
2	6	4	0.6

D Tophi

PLS-DA cross validation details:

Measure	1 comps	2 comps	3 comps	4 comps	5 comps
Accuracy	0.7	0.55	0.6	0.65	0.65
R2	0.66183	0.90699	0.98699	0.99713	0.99971
Q2	0.094167	-0.10215	-0.18112	-0.15384	-0.15364

The OOB error is 0.3

	N	Y	class.error
N	14	1	0.0667
Y	5	0	1.0

Supplementary Figure 2

A

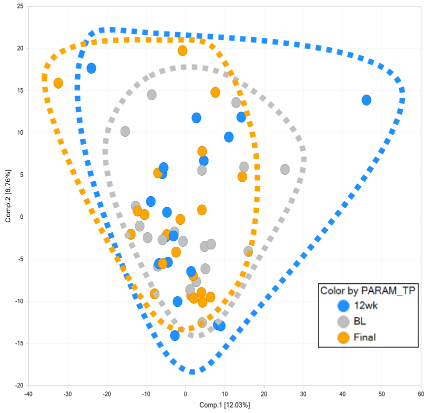

B

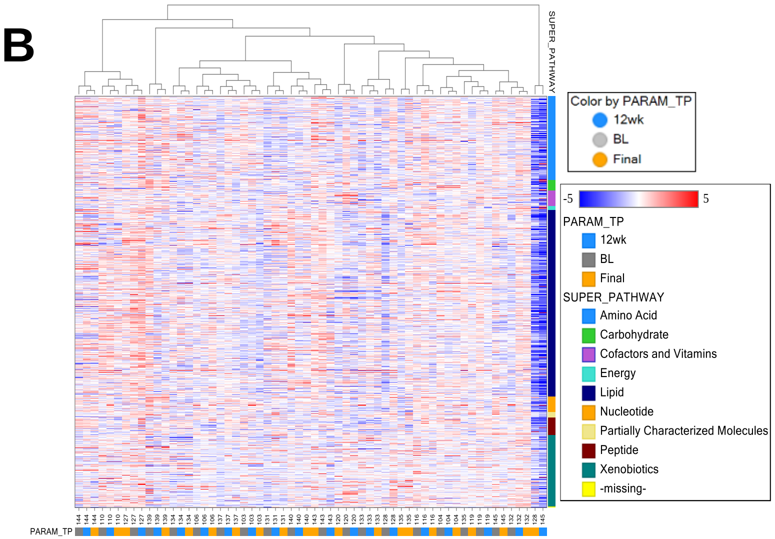

C

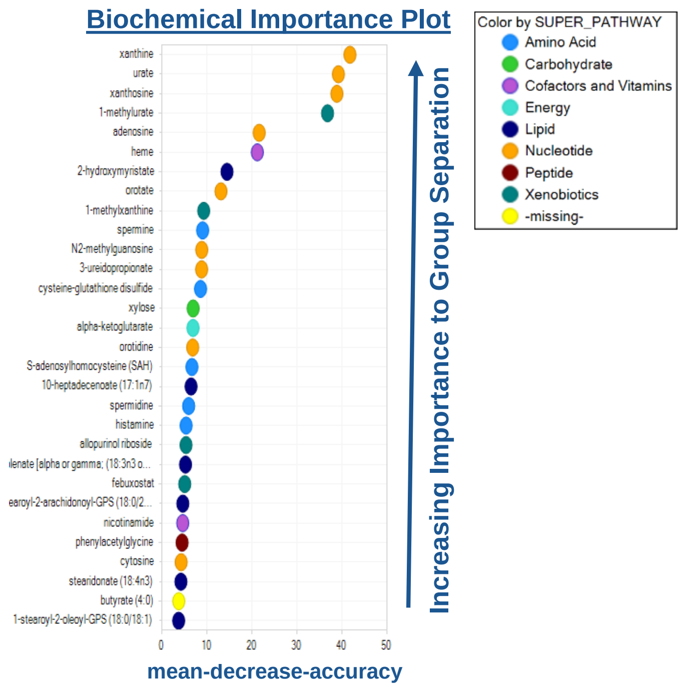

D

	BL	12 wks	Final	Class Error
BL	15	4	1	0.250
12wks	4	6	9	0.684
Final	3	8	10	0.523
Predictive accuracy = 52%				

Random Forest Confusion Matrix

Supplementary Figure 3

A

Sub Pathway	Biochemical Name	Fold of Change: ANOVA Contrasts: 12wk / BL	Fold of Change: ANOVA Contrasts: Final / BL	Fold of Change: ANOVA Contrasts: Final / 12wk	Fold of Change: Two-Way ANOVA: Group Main Effect
Purine Metabolism, (Hypo)Xanthine/Inosine containing	inosine	0.77	0.77	0.99	
	hypoxanthine	0.93	0.81	0.87	
	xanthine	3.00	3.00	1.00	
	xanthosine	3.89	3.44	0.88	
	N1-methylinosine	0.96	0.94	0.98	
	urate	0.76	0.74	0.97	
	allantoin	0.79	0.76	0.95	
Pyrimidine Metabolism, Orotate containing	dihydroorotate	1.21	1.13	0.93	
	orotate	3.24	3.10	0.96	
	orotidine	1.82	2.18	1.20	
Drug - Metabolic	metformin	1.10	0.59	0.54	
	atorvastatin (lipitor)	0.60	0.29	0.48	
	o-hydroxyatorvastatin	0.59	0.31	0.54	
	gemfibrozil	0.78	0.64	0.81	
	allopurinol	29.94	38.08	1.27	
	allopurinol riboside	2.15	2.65	1.23	
	oxypurinol	3.10	3.37	1.09	
	febuxostat	85.65	59.22	0.69	
Xanthine Metabolism	caffeine	1.32	1.00	0.76	
	paraxanthine	1.14	1.06	0.93	
	theobromine	1.29	1.25	0.97	
	theophylline	1.21	1.14	0.94	
	1-methylurate	0.29	0.26	0.88	
	7-methylurate	1.04	0.75	0.72	
	1,3-dimethylurate	0.74	0.83	1.13	
	1,7-dimethylurate	0.80	0.79	0.99	
	3,7-dimethylurate	1.35	1.19	0.88	
	1,3,7-trimethylurate	0.95	0.76	0.80	
	1-methylxanthine	2.13	2.47	1.16	
	3-methylxanthine	1.17	1.23	1.05	
	7-methylxanthine	1.45	1.57	1.09	
	5-acetylamino-6-amino-3-methyluracil	0.91	0.74	0.81	
	5-acetylamino-6-formylamino-3-methyluracil	1.16	0.79	0.68	

B

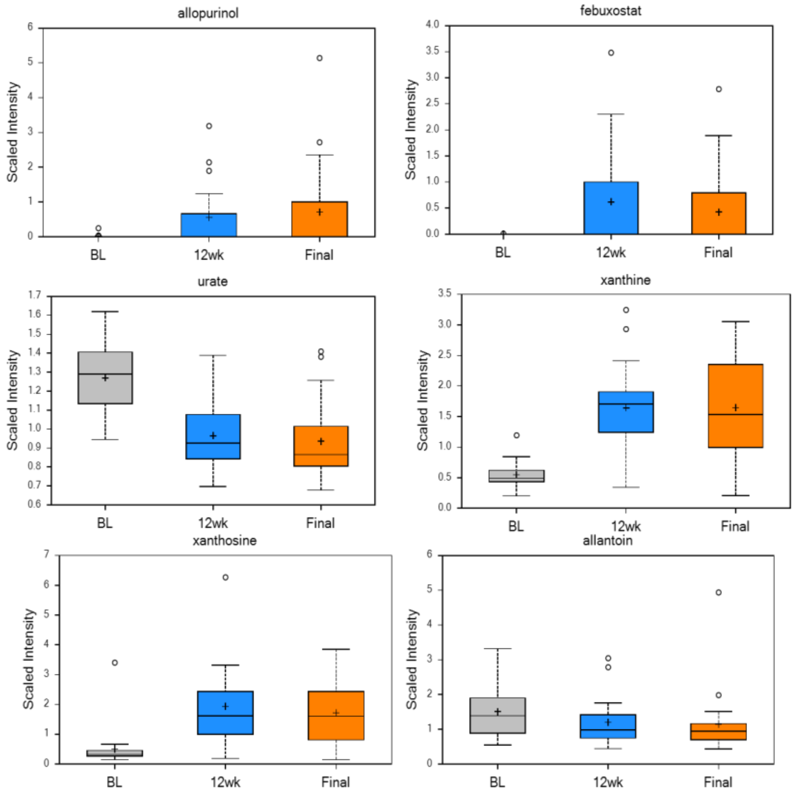

Supplementary Figure 4

A

Sub Pathway	Biochemical Name	Fold of Change			
		ANOVA Contrasts			Two-Way ANOVA
		12wk / BL	Final / BL	Final / 12wk	Group Main Effect
Secondary Bile Acid Metabolism	deoxycholate	0.94	0.97	1.04	
	deoxycholic acid (12 or 24)-sulfate*	1.37	0.83	0.61	
	deoxycholic acid glucuronide	1.09	0.73	0.67	
	glycodeoxycholate	1.53	1.79	1.18	
	taurodeoxycholate	0.85	1.21	1.43	
	taurodeoxycholic acid 3-sulfate	1.51	1.00	0.66	
	lithocholate sulfate (1)	1.09	0.65	0.59	
	glycolithocholate	1.34	1.24	0.92	
	glycolithocholate sulfate*	1.99	0.85	0.43	
	taurolithocholate 3-sulfate	1.15	0.64	0.56	
	ursodeoxycholate	0.75	1.09	1.46	
	isoursodeoxycholate	0.54	0.52	0.96	
	glycoursodeoxycholate	0.80	1.16	1.46	
	glycoursodeoxycholic acid sulfate (1)	1.81	1.03	0.57	
	tauroursodeoxycholate	0.39	0.52	1.36	
	hyocholate	0.73	1.24	1.69	
	glycohyocholate	2.11	1.60	0.76	
	taurohyocholate*	0.88	1.87	2.12	
	glycocholenate sulfate*	1.04	0.90	0.87	
	taurocholenate sulfate*	0.87	0.83	0.96	
	3b-hydroxy-5-cholenoic acid	1.10	0.96	0.87	
	glycodeoxycholate 3-sulfate	2.00	1.07	0.53	
	taurochenodeoxycholic acid 3-sulfate	1.25	1.00	0.80	
	glycodeoxycholate glucuronide (1)	1.36	0.89	0.65	

B

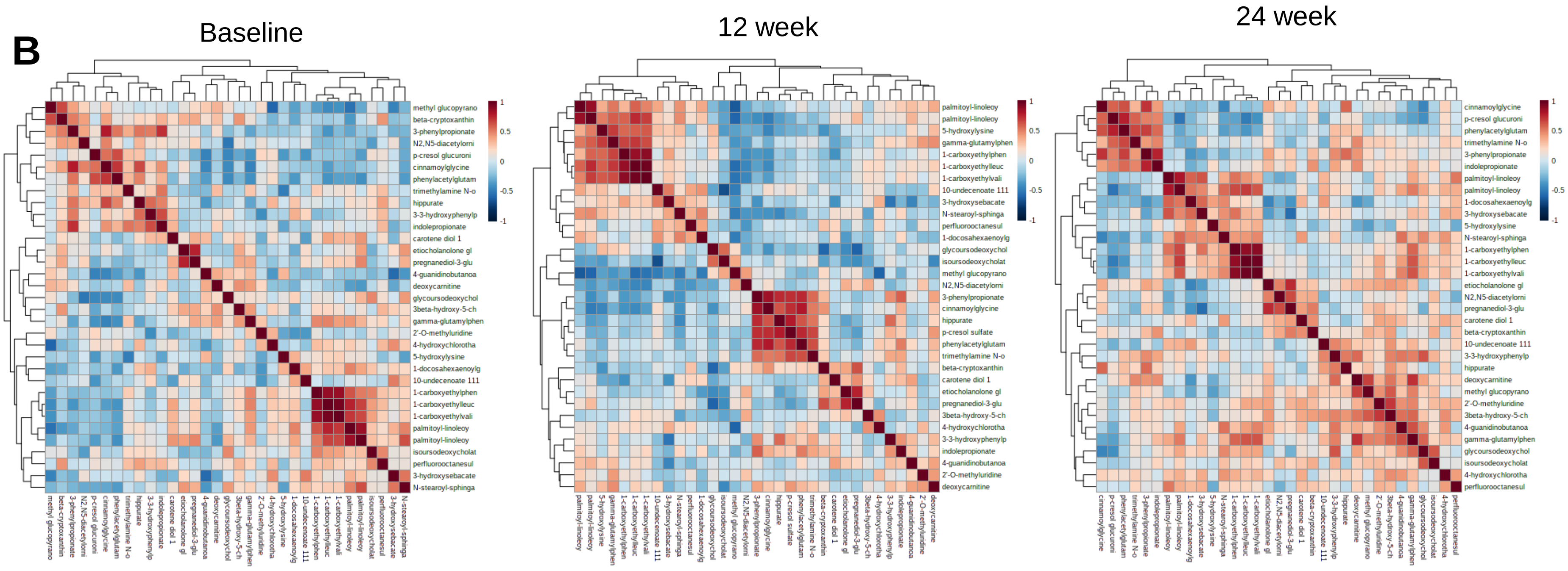

Supplementary Figure 5

A

Super Pathway	Sub Pathway	Biochemical Name	Fold of Change — ANOVA Contrasts: 12wk / BL	Final / BL	Final / 12wk	Two-Way ANOVA: Group Main Effect
Cofactors and Vitamins	Nicotinate and Nicotinamide Metabol	quinolinate	1.13	1.08	0.95	
		nicotinamide	0.98	0.62	0.63	
		1-methylnicotinamide	1.46	0.80	0.55	
		trigonelline (N'-methylnicotinate)	1.07	0.97	0.91	
		N1-Methyl-2-pyridone-5-carboxamide	0.92	0.81	0.89	
		N1-Methyl-4-pyridone-3-carboxamide	0.94	0.84	0.90	
	Riboflavin Metabolism	riboflavin (Vitamin B2)	0.78	0.56	0.71	
	Pantothenate and CoA Metabolism	pantoate	2.22	0.92	0.42	
		pantothenate	1.05	0.98	0.94	
	Ascorbate and Aldarate Metabolism	ascorbic acid 2-sulfate	0.88	0.85	0.97	
		ascorbic acid 3-sulfate*	1.01	0.89	0.88	
		threonate	0.96	0.90	0.94	
		oxalate (ethanedioate)	0.95	0.86	0.91	
		gulonate*	0.99	0.96	0.96	
	Tocopherol Metabolism	alpha-tocopherol	0.99	1.03	1.04	
		delta-tocopherol	0.93	0.92	0.99	
		gamma-CEHC	0.98	0.77	0.79	
		gamma-CEHC glucuronide*	0.90	0.64	0.72	
		alpha-CEHC glucuronide*	0.56	0.53	0.95	
		alpha-CEHC sulfate	0.69	0.62	0.91	
		delta-CEHC	1.00	0.88	0.88	
		delta-CEHC glucuronide*	0.87	0.83	0.94	
		gamma-CEHC sulfate	0.96	1.13	1.18	
		gamma-tocopherol/beta-tocopherol	0.98	0.92	0.94	
	Hemoglobin and Porphyrin Metabolis	heme	0.58	0.19	0.34	
		bilirubin (Z,Z)	1.02	1.04	1.02	
		bilirubin (E,E)*	1.02	1.06	1.04	
		bilirubin (E,Z or Z,E)*	1.10	1.32	1.20	
		biliverdin	0.90	0.59	0.65	
		I-urobilinogen	1.22	1.60	1.31	
		L-urobilin	1.24	0.39	0.32	
	Thiamine Metabolism	thiamin (Vitamin B1)	1.00	1.00	1.00	
	Vitamin A Metabolism	retinol (Vitamin A)	0.99	1.05	1.05	
		carotene diol (1)	1.03	1.19	1.15	
		carotene diol (2)	1.03	1.14	1.11	
		carotene diol (3)	0.99	1.11	1.12	
		beta-cryptoxanthin	1.16	1.01	0.87	
		retinal	0.88	0.77	0.87	
	Vitamin B6 Metabolism	pyridoxine (Vitamin B6)	1.86	0.65	0.35	
		pyridoxal	1.14	0.74	0.65	
		pyridoxate	0.76	0.47	0.62	

B

Sub Pathway	Biochemical Name	Fold of Change — ANOVA Contrasts: 12wk / BL	Final / BL	Final / 12wk	Two-Way ANOVA: Group Main Effect
Polyamine Metabolism	N-acetylputrescine	1.07	1.12	1.05	
	N-acetyl-isoputreanine	1.10	1.08	0.98	
	spermidine	1.15	0.75	0.65	
	(N(1) + N(8))-acetylspermidine	1.04	0.99	0.95	
	acisoga	1.20	0.97	0.81	
	spermine	0.99	0.49	0.49	
	5-methylthioadenosine (MTA)	1.14	1.10	0.96	
	4-acetamidobutanoate	0.99	1.06	1.07	
